# Sociodemographic, health behavioral, and disease history risk factors for dementia in older adults: a population-based cross-sectional study in Guangzhou, China

**DOI:** 10.3389/fpubh.2025.1640089

**Published:** 2025-08-29

**Authors:** Xinyue Huang, Cuibing Li, Guixian Tao, Yi Zhong, Jiahui Li, Tinglong Chen, Jichuan Shen

**Affiliations:** ^1^School of Public Health, Sun Yat-sen University, Guangzhou, China; ^2^School of Public Health, Guangzhou Medical University, Guangzhou, China; ^3^Guangzhou Center for Disease Control and Prevention, Guangzhou Health Supervision Institute, Guangzhou, China

**Keywords:** dementia, older adults, prevalence, risk factors, cross-sectional study

## Abstract

**Objective:**

This study aimed to estimate the prevalence of dementia and its associated risk factors among older adults aged 65 years and above in Guangzhou, China.

**Methods:**

We conducted a community-based cross-sectional study involving 2,463 residents aged 65 years or older in Guangzhou, using a multi-stage whole-population random sampling method. Cognitive function was assessed using the Mini-Mental State Examination (MMSE), with cutoff points adjusted by education level. Trained investigators collected information on demographic characteristics, health behavioral factors, and medical history through face-to-face interviews. Univariate analyses and multivariate logistic regression were performed to identify factors associated with dementia.

**Results:**

The prevalence of dementia was 14.94% among participants aged 65 and above, increasing from 13.30 to 34.58% across different age groups. Multivariate analysis showed that frequent falls (≥3 falls within the past year vs. no falls: *OR* = 2.72, 95% *CI*: 1.27–5.81, *p* < 0.05), older age (≥85 years vs. 65–74 years: *OR* = 2.62, 95% *CI*: 1.66–4.13, *p* < 0.001), depression (*OR* = 2.61, 95% *CI*: 1.36–5.00, *p* < 0.05) had the most significant associations with dementia risk. Lower educational attainment, prolonged sleep duration (≥8 h), lack of cognitive activity and physical activity, depression, as well as a history of stroke, also showed statistical differences.

**Conclusion:**

Dementia is highly prevalent among older adults in Guangzhou, posing a substantial public health challenge. Our findings emphasize the need for early identification and targeted interventions, including the promotion of physical and cognitive activities, fall prevention strategies, and better management of chronic conditions such as stroke, depression, and hearing loss, to reduce dementia risk and burden in aging communities.

## 1 Introduction

Dementia, a progressive, irreversible neurodegenerative disease, has emerged as a critical public health concern due to its growing prevalence and associated substantial health and socioeconomic burdens (1, 2). This issue has increasingly attracted widespread attention across the public health domain in recent years. The World Health Organization (WHO) reports that 55 million people were estimated to have dementia globally in 2019, and the figure is projected to rise to 139 million by 2050 (3). With the aging of the global population, dementia is becoming one of the leading causes of death worldwide (4). The rising costs of dementia are creating more health and social challenges for people in urban and rural areas across China (5). The most effective interventions to reduce dementia risk should be applied in the early stages of the disease (6, 7).

China has the largest dementia population in the world, with a high prevalence and mortality rate (8). The prevalence of Alzheimer’s disease increased significantly from 3.68 million in 1990 to 10 million in 2020 (9). The China Alzheimer’s Disease Report 2024 reported that Guangdong Province ranked sixth nationwide in age-standardized prevalence rate of AD and other dementias (836.4/100,000 population) (10). The prevalence of dementia and distribution of associated risk factors in Guangzhou (the capital of Guangdong Province) remain unclear.

The causes of dementia are still unclear, and epidemiologically, dementia arises from multifactorial interactions among risk factors (11). Previous studies have explored factors influencing the onset and prevalence of all-cause dementia, comprising two main categories: non-modifiable risk factors such as genetics and age, and modifiable risk factors encompassing health behaviors, medical history, dietary patterns, educational level, social activities, and living environment (5). Some studies propose that age, stroke, hypertension, diabetes, and genetics as independent risk factors for dementia, while other factors show potential associations with dementia, their causal relationships require validation through population-based studies (7, 12, 13). Despite advances in understanding its risk factors, few pieces of evidence from southern Chinese populations, such as those in Guangzhou, are hindering the development of targeted interventions. Comprehensive epidemiological investigations are required to establish accurate population-level estimates and require urgent updating to inform evidence-based public health strategies.

This study aims to estimate the prevalence of dementia by conducting a large-scale population-based epidemiological survey of sociodemographic characteristics of people aged ≥65 years old in the Guangzhou community, and to investigate the relationship between sociodemographic factors, health behavioral factors, and medical history, and cognitive decline in dementia.

## 2 Materials and methods

### 2.1 Study design and participants

This cross-sectional study was conducted in Guangzhou, China. From June 2023 to December 2024, based on the Free Physical Examination Program for people aged 65 years and above, a multistage whole-population random sampling method was used to randomly select 6 out of 11 districts in Guangzhou, and 2 townships/streets were randomly selected in each district using the whole-population random sampling method, and each township/street randomly selected 200 cases of permanent residents ≥65 years old who had established health management records in local community hospitals for on-site questionnaire survey. Inclusion criteria: (a) local residents who have lived in the area for more than half a year (b) aged ≥65 years (c) voluntarily cooperated with the survey work of this project and signed the informed consent form. Exclusion criteria: (a) unable to complete the questionnaire survey (b) incomplete information in the questionnaire survey.

### 2.2 Procedure

Study data were collected by trained investigators through face-to-face interviews and cognitive testing through means of a questionnaire. The questionnaire included information on demographic characteristics (e.g., gender, age, education, occupation, living status, marital status), health behaviors (e.g., smoking, alcohol consumption, physical activity, cognitive activity), and medical history (e.g., hypertension, diabetes, stroke). The investigators checked the completeness of the questionnaires on site, and finally 2,463 valid questionnaires were analyzed and included in this study.

### 2.3 Cognitive function assessment

The cognitive functioning of the study participants was assessed by the Mini-Mental State Examination (MMSE). This study used a modified Chinese version of the MMSE, which has good validity and reliability in patients with dementia and the general population (14, 15). MMSE evaluates dementia through five domains, including orientation, memory, attention, computation, and language abilities (16). A total score of 30 points was used to assess cognitive impairment categorized by different education levels, which initially suggested the presence of dementia (17). Dementia was diagnosed based on the fourth edition of the Diagnostic and Statistical Manual of Mental Disorders (DSM-IV) (18). Dementia was identified using education-adjusted MMSE cutoffs: ≤17 for illiterate participants, ≤20 for those with elementary education, and ≤24 for those with secondary education or above (14, 19).

### 2.4 Assessment of covariates

Baseline data of sociodemographic characteristics (gender, age, education, occupation, marital status, living status), health behavioral factors (alcohol consumption, smoking, physical exercise, Denture use, cognitive activity, sleep, falls), and medical history (e.g., hypertension, diabetes, dyslipidemia) were treated as covariates. Gender, age, and education were self-reported by the participants. Marital status was classified into married and others (including divorced, widowed, and never married). Living status was classified into living alone and with others. Alcohol consumption and smoking were categorized into three groups: never, former, and current. Physical activity is moderate-intensity aerobic activity (e.g., walking, brisk walking, housework, dancing) or vigorous-intensity aerobic activity (e.g., running, hiking, biking) for at least 150 min per week for more than 1 year (20). Cognitive activity is participation in at least one of the following activities (reading, writing, drawing, board games, mahjong activities, etc.) for more than 1 year, no less than 2 times per week, each time ≥30 min (21). Denture use was assessed by asking the participants, “Do you have dentures?” The options were “yes” and “no.” Difficulty initiating sleep (DIS) was defined as requiring ≥30 min to fall asleep on ≥3 nights per week (22). Nocturnal awakenings (NAs) were defined as experiencing ≥1 episode of nighttime awakening per week over the past month, with difficulty returning to sleep within 30 min (23). Sleep duration was assessed by asking participants how long they slept each night. Falls were assessed by asking participants whether they had fallen in the past year, how many times they had fallen, and whether they had been injured as a result of a fall. Depression in disease history is defined as a score of ≥5 on the Geriatric Depression Scale (GDS-15) (24). Other diseases in the medical history are based on self-reported medical history or results of medical examinations at community hospitals and medical records.

### 2.5 Statistical analysis

The participants were divided into dementia and cognitively normal groups to describe their basic characteristics. The chi-square test or *Fisher’s* exact test was used for univariate analyses. Multivariate analysis was performed using a backward stepwise binary logistic regression model (likelihood ratio test), and adjusted odds ratios (*ORs*) with 95% confidence intervals were calculated. Data analysis was performed using IBM SPSS Statistics for Windows (version 26.0; IBM Corp., Armonk, NY, USA). A two-tailed *p* < 0.05 was considered statistically significant.

### 2.6 Ethical statement

After the survey was completed, all data were stored anonymously without names or identifying information to protect participants’ confidentiality. For participants with impaired cognitive function, proxy respondents (family members or primary caregivers) assisted in completing the questionnaire. Written informed consent was obtained from all participants or their proxies after they were fully informed about the study. The present study was approved by the Ethics Committee of the Guangzhou Centre for Disease Control and Prevention (GZCDC-ECHR-2022P0042).

## 3 Result

### 3.1 Baseline sociodemographic characteristics of the study population

A total of 2,463 older adults were included in this study, of which 41.70% were male and 58.30% were female. In terms of age distribution, 1,744 participants (70.81%) were aged 65–74 years, 612 (24.85%) were aged 75–84 years, and 107 (4.34%) were aged 85 years or older. Regarding education level, 7.71% of the participants were illiterate, 53.23% had primary or junior high school education, and 39.06% attained high school or above. Most participants were married (78.68%), engaged in manual occupations before retirement (50.02%), and lived with others (92.08%) (Table 1).

**Table 1:** Sociodemographic characteristics of participants.

Characteristic	Variables	*N*	%
Gender	Male	1,027	41.70
	Female	1,436	58.30
Age (years)	65~	1,744	70.81
	75~	612	24.85
	85~	107	4.34
Education level	Illiterate	190	7.71
	Primary and junior high school	1,311	53.23
	High school or above	962	39.06
Occupation	Manual laborer	1,232	50.02
	Mental laborer	1,231	49.98
Marital status	Unmarried/divorced/widowed	525	21.32
	Married	1,938	78.68
Living status	Live alone	195	7.92
	With others	2,268	92.08

### 3.2 Prevalence of dementia in older adults

Among 2,463 participants, 14.94% (368/2,463) were diagnosed with dementia. Dementia prevalence increased with age, ranging from 13.30% (232/1,744) in the 65–74 years group to 34.58% (37/107) in those aged ≥85 years. Illiterate individuals exhibited the highest dementia prevalence (24.74%, 47/190), compared to 9.67% (93/962) in participants with high school or higher education. Manual workers had a higher prevalence (17.78%, 219/1,232) than mental workers (12.10%, 149/1,231). Unmarried/divorced/widowed individuals showed elevated prevalence (18.67%, 98/525) relative to married participants (13.93%, 270/1,938). Dementia prevalence was marginally higher in those living alone (17.44%, 34/195) compared to cohabiting individuals (14.73%, 334/2,268) (Table 2).

**Table 2:** Prevalence of dementia by sociodemographic characteristics.

Variables	Total	Normal	Dementia	$\chi^{2}$	*p*-value
Overall	2,463	2,095 (85.06)	368 (14.94)		
Gender				0.03	0.859
Male	1,027	872 (84.91)	155 (15.09)		
Female	1,436	1,223 (85.17)	213 (14.83)		
Age (years)				36.89	<0.001^*^
65~	1,744	1,512 (86.70)	232 (13.30)		
75~	612	513 (83.82)	99 (16.18)		
85~	107	70 (65.42)	37 (34.58)		
Education level				41.59	<0.001^*^
Illiterate	190	143 (75.26)	47 (24.74)		
Primary and junior high school	1,311	1,083 (82.61)	228 (17.39)		
High school or above	962	869 (90.33)	93 (9.67)		
Occupation				15.59	<0.001^*^
Manual laborer	1,232	1,013 (82.22)	219 (17.78)		
Mental laborer	1,231	1,082 (87.90)	149 (12.10)		
Marital status				7.29	0.007^*^
Unmarried/Divorced/Widowed	525	427 (81.33)	98 (18.67)		
Married	1,938	1,668 (86.07)	270 (13.93)		
Living status				1.04	0.309
Live alone	195	161 (82.56)	34 (17.44)		
With others	2,268	1,934 (85.27)	334 (14.73)		

*p < 0.05.

### 3.3 Association between sociodemographic factors and dementia

The analysis revealed that age, education level, occupation, and marital status were significant factors influencing Dementia risk (*p* < 0.05, Table 2). Specifically, advanced age (≥85 years), lower education (illiteracy), manual occupations, and unmarried/divorced/widowed status were strongly associated with higher Dementia prevalence. Gender and living status showed no statistically significant associations (*p* > 0.05).

### 3.4 Association between health behavioral factors and dementia

The analysis identified physical exercise, cognitive activity, sleep duration, history of falls in the past year, fall-related injuries, and frequency of falls as significant behavioral factors associated with dementia (*p* < 0.05, Table 3). Alcohol consumption, smoking, denture use, difficulty initiating sleep, and Nocturnal awakenings were not significantly associated with dementia (*p* > 0.05, Supplementary Table S1).

**Table 3:** Prevalence of dementia by health behavioral factors.

Variables	Total	Normal	Dementia	$\chi^{2}$	*p*-value
Alcohol consumption				4.93	0.085
Never drank alcohol	2,014	1,700 (84.41)	314 (15.59)		
Currently drink alcohol	315	281 (89.21)	34 (10.79)		
Have quit drinking alcohol	134	114 (85.07)	20 (14.93)		
Smoking				2.62	0.270
Never smoked	1,877	1,595 (84.98)	282 (15.02)		
Currently smoke	321	267 (83.18)	54 (16.82)		
Have quit smoking	265	233 (87.92)	32 (12.08)		
Physical exercise				18.69	<0.001^*^
With	2,282	1,961 (85.93)	321 (14.07)		
Without	181	134 (74.03)	47 (25.97)		
Cognitive activity				16.64	<0.001^*^
With	902	802 (88.91)	100 (11.09)		
Without	1,561	1,293 (82.83)	268 (17.17)		
Sleep duration				32.57	<0.001^*^
<6 h	339	286 (84.37)	53 (15.63)		
6-8 h	1,657	1,450 (87.51)	207 (12.49)		
≥8 h	467	359 (76.87)	108 (23.13)		
History of falls in the past year				6.39	0.011^*^
Yes	274	219 (79.93)	55 (20.07)		
No	2,189	1,876 (85.70)	313 (14.30)		
Fall frequency within the past year				10.19	0.006^*^
0	2,191	1,878 (85.71)	313 (14.29)		
1 ~ 2	236	1,192 (81.36)	44 (18.64)		
≥3	36	25 (69.44)	11 (30.56)		
Fall-related injuries				5.16	0.023^*^
With	178	141 (79.21)	37 (20.79)		
Without	2,285	1,954 (85.51)	331 (14.49)		

*p < 0.05.

### 3.5 Association between medical history and dementia

The analysis revealed that a history of stroke, depression, hearing impairment, hypertension, diabetes, and dyslipidemia were significantly associated with dementia (*p* < 0.05, Table 4). Among these, stroke, depression, and hearing impairment exhibited the strongest associations with increased dementia risk. Hypertension (16.96% vs. 13.18%, *p* = 0.009) and diabetes (18.04% vs. 14.15%, *p* = 0.030) also showed modest but statistically significant associations. Chronic diseases such as gout, chronic lung disease, and rheumatoid arthritis were not significantly associated with dementia (*p* > 0.05, Supplementary Table S2).

**Table 4:** Prevalence of dementia by medical history.

Variables	Total	Normal	Dementia	$\chi^{2}$	*p*-value
Hypertension				6.90	0.009^*^
Yes	1,150	955 (83.04)	195 (16.96)		
No	1,313	1,140 (86.82)	173 (13.18)		
Diabetes				4.72	0.030^*^
Yes	499	409 (81.96)	90 (18.04)		
No	1,964	1,689 (85.85)	278 (14.15)		
Dyslipidemia				3.85	0.050^*^
Yes	189	170 (89.95)	19 (10.05)		
No	2,274	1,925 (84.65)	349 (15.35)		
Stroke				19.72	<0.001^*^
Yes	98	68 (69.39)	30 (30.61)		
No	2,365	2,027 (85.71)	338 (14.29)		
Hearing impairment				8.05	0.005^*^
Yes	43	30 (69.77)	13 (30.23)		
No	2,420	2,065 (85.33)	355 (14.67)		
Depression				16.15	<0.001^*^
Yes	48	31 (64.58)	17 (35.42)		
No	2,415	2,064 (85.47)	351 (14.53)		

*p<0.05.

### 3.6 Logistic regression analysis of dementia risk factors

A binary logistic regression analysis was conducted with dementia status (0 = no, 1 = yes) as the dependent variable, and 16 independent variables, including age, educational level, occupation, marital status, physical exercise, cognitive activity, sleep duration, history of falls in the past year, fall-related injuries, frequency of falls, hypertension, diabetes, dyslipidemia, stroke, hearing impairment, and depression. To assess multicollinearity among the independent variables, variance inflation factors (VIF) were calculated. The VIF for history of falls in the past year (12.59) and frequency of falls (10.22) indicated severe multicollinearity. Since the VIF for history of falls was higher and frequency of falls was deemed more informative for predicting dementia risk, the former was excluded to reduce the impact of multicollinearity on the model; following this adjustment, all remaining variables exhibited VIF < 5, indicating acceptable levels of collinearity (Supplementary Table S3). The Hosmer-Lemeshow test indicated satisfactory model fit ( $\chi^{2}$ = 8.664, *df* = 7, *p* = 0.278), suggesting that predicted probabilities agreed well with observed outcomes (Supplementary Table S4).

Multivariate logistic regression identified several significant predictors of dementia (Table 5). Compared to individuals aged 65–74, those aged 85 and above had significantly higher odds of dementia (*OR* = 2.62, 95% *CI*: 1.66–4.13, *p* < 0.001). Higher educational attainment (high school or above) was associated with lower odds (*OR* = 0.43, 95% *CI*: 0.28–0.65, *p* < 0.001). Regular physical exercise (*OR* = 0.68, 95% *CI*: 0.46–0.99, *p* = 0.046) and cognitive activity (*OR* = 0.72, 95% *CI*: 0.55–0.93, *p* = 0.012) were protective factors. In contrast, longer sleep duration (≥ 8 h) was linked to increased risk (*OR* = 1.87, 95% *CI*: 1.43–2.45, *p* < 0.001). Experiencing more than three falls in the past year was also associated with significantly elevated odds of dementia (*OR* = 2.72, 95% *CI*: 1.27–5.81, *p* = 0.010). Additionally, depression (*OR* = 2.61, 95% *CI*: 1.36–5.00, *p* = 0.004), stroke (*OR* = 1.81, 95% *CI*: 1.12–2.93, *p* = 0.015), and hearing impairment (*OR* = 2.30, 95% *CI*: 1.14–4.66, *p* = 0.021) were independent risk factors.

**Table 5:** Multivariate logistic regression analysis of factors associated with dementia.

Variable	*β*	*SE*	*Waldχ^2^*	*OR (95% CI)*	*p*-value
Age					
65–74	Ref.				
75–84	0.064	0.136	0.226	1.07 (0.82–1.39)	0.635
≥85	0.963	0.233	17.101	2.62 (1.66–4.13)	<0.001^*^
Education level					
Illiterate	Ref.				
Primary and junior high school	−0.207	0.194	1.138	0.81 (0.56–1.19)	0.813
High school or above	−0.855	0.214	15.930	0.43 (0.28–0.65)	<0.001^*^
Physical exercise					
Without	Ref.				
With	−0.391	0.196	3.974	0.68 (0.46–0.99)	0.046^*^
Cognitive activity					
Without	Ref.				
With	−0.331	0.133	6.25	0.72 (0.55–0.93)	0.012^*^
Sleep duration					
6–8 h	Ref.				
<6 h	0.076	0.174	0.191	1.08 (0.77–1.52)	0.662
≥8 h	0.626	0.139	20.375	1.87 (1.43–2.45)	<0.001^*^
Fall frequency within the past year					
0	Ref.				
1 ~ 2	0.256	0.187	1.877	1.29 (0.90–1.87)	0.171
≥3	1.000	0.387	6.657	2.72 (1.27–5.81)	0.010^*^
Depression					
No	Ref.				
Yes	0.959	0.332	8.340	2.61 (1.36–5.00)	0.004^*^
Stroke					
No	Ref.				
Yes	0.596	0.245	5.902	1.81 (1.12–2.93)	0.015^*^
Hearing impairment					
No	Ref.				
Yes	0.833	0.360	5.343	2.30 (1.14–4.66)	0.021^*^

*p<0.05.

## 4 Discussion

In this cross-sectional study conducted in Guangzhou, China, we found that the prevalence of dementia among older adults aged 65 years or older was 14.90%, ranging between 13.30 and 34.58% in different age groups. Sociodemographic factors of advanced age and low education level, health behavioral factors of long sleep duration, multiple falls in a year, lack of cognitive activity and physical exercise, and medical history of depression, stroke, and hearing impairment were risk factors for the development of dementia.

In this study, the prevalence of dementia in the older population was 14.94% based on the MMSE scale screening. This result is both consistent with several studies in China and specific due to differences in screening criteria, adjustment for educational level, and population characteristics. Several studies have reported on the prevalence of dementia in China, but estimates vary: the prevalence of dementia ranges from 5.00 to 7.70% for those aged 60 years and over, and from 2.00 to 13.00% for those aged 65 years and older (9). A multi-city cross-sectional study conducted in China between 2008 and 2009 reported a dementia prevalence of 5.14% (95% *CI*: 4.71–5.57%) among individuals aged 65 years and older (25); A meta-analysis of 96 studies showed that the overall prevalence of dementia among people aged 60 years and older in China was 5.30% (95% *CI*:4.30–6.30%) (26). Two studies conducted in Hong Kong community populations, also using the MMSE as a screening tool, reported dementia prevalence rates of 18.90% (27) and 13.90% (28) respectively, which were similar to the results of our study. It is worth noting that the MMSE, as a brief cognitive screening tool, often has different score thresholds in different studies depending on the level of education, which is an important factor contributing to the variation in prevalence estimates (29). In this study, the MMSE scale rating scale by education level was used, which helped to improve the diagnostic sensitivity of the low-education population (15). However, even so, the prevalence rate of 14.94% is still at a high level, suggesting that the burden of dementia risk may be more severe in the region, which may be related to several factors such as demographics, lifestyle, and the burden of chronic disease.

In our study, age, education level, physical exercise, cognitive activity, sleep duration, fall frequency within 1 year, stroke, hearing impairment, and depression were associated with dementia risk. Age is one of the non-modifiable risk factors. Aging causes the brain to accumulate pathological hallmarks of dementia, such as amyloid plaques, tau protein tangles, and vasculopathy. These age-related neurobiological changes collectively contribute to an elevated risk of dementia development (30). The study also identified 8 modifiable risk factors, including education level, physical exercise, and cognitive activity, among others. Higher education and complex occupations may contribute to increasing an individual’s level of cognitive reserve, as supported by our study and consistent with previous findings (31). Considering the association between educational attainment and dementia, older adults should prioritize lifelong learning, especially in rural areas with limited educational resources, and enhanced cognitive reserve can reduce the risk of dementia.

Regarding health behaviors, we identified physical exercise and cognitive activity as significant modifiable protective factors associated with a reduced risk of dementia. A meta-analysis suggests that structured physical exercise and cognitive activity confer neuroprotective benefits against dementia, possibly through a reduction in *β*-amyloid, an improvement in the cerebrovascular system and blood flow, as well as antioxidant and inflammatory processes in the brain (32). A large longitudinal observational study in Hong Kong, China, demonstrated that adults aged 65 years and older who read, play games, or gamble more frequently exhibited a reduced risk of developing dementia (*OR* = 0.7, 95% *CI*: 0.6–0.8), which is consistent with our findings (33). Our study emphasizes the importance of promoting modifiable lifestyle factors in older populations and promoting policy development for regular physical activity programs to strengthen dementia prevention strategies and improve clinical care for older adults. In contrast to previous studies, our study reveals that long sleep duration increases the risk of dementia, while finding no association between short sleep duration and dementia risk, possibly because variations in association strength across age groups were not explicitly considered, resulting in the masking of this association (34). The findings also suggest that multiple falls (≥3 falls in the past year) (*OR* = 2.72, 95% *CI*: 1.27–5.81, *p* = 0.010) are associated with the risk of developing dementia. While The Lancet’s 2024 report does not explicitly include falls among the 14 modifiable dementia risk factors, repeated falls may increase the risk of dementia by accelerating *β*-amyloid deposition through traumatic brain injury (TBI) or chronic inflammatory responses. This finding may be associated with the pathological mechanism of “vascular damage—inflammation—neurodegeneration axis” described in the report (35). Several cohort studies indicate a potential bidirectional association between falls and dementia (36).

Among the associations between chronic diseases and dementia, it was found that hypertension, diabetes, dyslipidemia, and stroke were found to be associated with dementia. Following multivariable adjustment for covariates (e.g., age and educational level, and so on), only stroke (*OR* = 1.81, 95% *CI*: 1.12–2.93) remained significantly associated with dementia, possibly due to confounding by other strongly associated factors (e.g., age, stroke, or depression). Several longitudinal studies have previously implicated control of diabetes and hypertension in reducing the risk of dementia, a possible explanation for these divergent findings is that the association between dementia risk and chronic conditions exhibits age-dependent heterogeneity (37, 38). Recent studies have demonstrated that the association between hypertension and dementia risk diminishes with advancing age (39). Furthermore, the long-term risk of dementia may be attributed to other vascular risk factors, such as stroke-induced focal brain injury with accelerated vascular cognitive impairment (40). Multiple meta-analyses have consistently demonstrated that stroke approximately doubles the risk of dementia, suggesting that stroke is an independent risk factor for dementia, consistent with the results of our study (41, 42).

Studies have also identified an association between hearing impairment and dementia, with potential mechanisms including hearing impairment-induced social isolation and depression, collectively increasing cognitive load and accelerating dementia progression. Additionally, inflammation or neurodegeneration in the auditory pathway may share common pathologic mechanisms with dementia (43). Prior research has suggested that hearing aids can improve short-term and semantic memory performance, while our findings endorse integrating hearing assessments and hearing aid interventions into geriatric care programs targeted at addressing hearing impairment as a modifiable risk factor for dementia (44). In this study, we found that depression (*OR* = 2.61, 95% *CI*: 1.36–5.00, *p* < 0.05) is an important risk factor for dementia, with depressed older adults having twice the risk of dementia compared to non-depressed counterparts, which is consistent with the trend observed in the random-effects meta-analysis results reported in The Lancet’s 2024 report (RR = 2.25, 95% CI: 1.69–2.98) (35). Such individuals exhibit more severe cognitive impairment, particularly in language skills and memory domains, a finding consistent with prior evidences (45). Screening and treating depression in older adults should be a priority, and dementia risk can be reduced through effective depression management and reduced social isolation.

Among the risk factors identified in this study, unmodifiable factors (e.g., age) are closely related to dementia, highlighting the need for early identification of high-risk groups (e.g., older adults). Community-based record-keeping with regular cognitive function monitoring can preserve a time window for subsequent intervention. Modifiable factors (e.g., insufficient cognitive activity and falls) are central to public health interventions: exposure risks can be reduced through strategic adjustments, and intervention costs are substantially lower than post-onset treatment burdens. Launched in 2017, the World-Wide FINGERS Network project uses multidomain lifestyle interventions—cognitive training, physical exercise, nutritional guidance, vascular risk control—to reduce cognitive decline and dementia risk (46, 47). Our findings provide a basis for promoting and optimizing public health interventions, with direct and far-reaching implications for dementia prevention and treatment.

This study has several important limitations. First, due to the cross-sectional design, causality and temporal relationships cannot be inferred; some “risk factors” (e.g., depression or falls) may instead be early manifestations of dementia rather than causes. Early dementia may initially manifest as non-cognitive symptoms (e.g., depression and apathy) or directly increase fall risk due to executive dysfunction and impaired spatial orientation (48, 49). Second, sociodemographic and lifestyle data were self-reported or proxy-reported, lacking objective validation (e.g., medical records), and may be subject to recall bias. Cognitive impairment itself could also impair accurate reporting, leading to overestimation or underestimation of associations. Third, reliance on the MMSE without gold-standard clinical confirmation introduces misclassification risk. Additionally, different diagnostic criteria can lead to significant misclassification bias; for example, DSM-IV, ICD-10, and CAMDEX criteria yield noticeably different dementia diagnoses in the same population. Fourth, our sample was drawn from communities in Guangzhou, which may limit generalizability to other Chinese regions or ethnic groups. Due to systemic differences between Guangzhou residents and typical rural populations (urbanization and healthcare access can affect both risk profiles and detection), the study results may not apply to rural residents. Fifth, the study did not control for social determinants (e.g., income, access to health services), which are recognized as important factors influencing cognitive decline, potentially limiting the generalizability of the findings.

Future studies should use a prospective cohort design to clarify the temporal sequence of variables with long-term follow-up. Objective data from electronic health records and wearable devices could enhance report accuracy. Additional relevant factors should be incorporated for comprehensive analysis, and research should be extended to rural settings to validate the generalizability of the findings.

## 5 Conclusion

In conclusion, dementia represents a significant health and social challenge in Guangzhou. Our study found a prevalence of 14.94% among individuals aged 65 years and older, with rates increasing from 13.30 to 34.58% across different age groups. This prevalence is notably higher than national estimates, which range from 2.00 to 13.00% for the same age group. The risk factors associated with higher dementia prevalence included advanced age, low education level, prolonged sleep duration, multiple falls within a year, lack of cognitive and physical activities, and medical histories of depression, stroke, and hearing impairment. These findings underscore the urgent need for targeted public health interventions in Guangzhou, focusing on modifiable risk factors and early detection strategies. Implementing community-based screening programs and promoting educational and physical activities among older adults could be effective measures to mitigate the growing burden of dementia in the region.

## Data Availability

The original contributions presented in the study are included in the article, further inquiries can be directed to the corresponding author.
