# Supplementary material for: Sociodemographic, health behavioral, and disease history risk factors for dementia in older adults: a population-based cross-sectional study in Guangzhou, China
Source: Front Public Health. 2025 Aug 29;13:1640089. doi: 10.3389/fpubh.2025.1640089 (PMC12425764; doi:10.3389/fpubh.2025.1640089)
Supplement: Supplementary file 1 [file Table_1.docx]

Supplementary Material

# Supplementary Tables

**TABLE S1 Prevalence of dementia by health behavioral factors.**

| Variables | Total | Normal | Dementia | $\chi^{2}$ | *P*-value |
| --- | --- | --- | --- | --- | --- |
| **Alcohol consumption** |  |  |  | 4.93 | 0.085 |
| Never drank alcohol | 2,014 | 1,700 (84.41) | 314 (15.59) |  |  |
| Currently drink alcohol | 315 | 281 (89.21) | 34 (10.79) |  |  |
| Have quit drinking alcohol | 134 | 114 (85.07) | 20 (14.93) |  |  |
| **Smoking** |  |  |  | 2.62 | 0.270 |
| Never smoked | 1,877 | 1,595 (84.98) | 282 (15.02) |  |  |
| Currently smoke | 321 | 267 (83.18) | 54 (16.82) |  |  |
| Have quit smoking | 265 | 233 (87.92) | 32 (12.08) |  |  |
| **Physical exercise** |  |  |  | 18.69 | ＜0.001^*^ |
| With | 2,282 | 1,961 (85.93) | 321 (14.07) |  |  |
| Without | 181 | 134 (74.03) | 47 (25.97) |  |  |
| **Cognitive activity** |  |  |  | 16.64 | ＜0.001^*^ |
| With | 902 | 802 (88.91) | 100 (11.09) |  |  |
| Without | 1,561 | 1,293 (82.83) | 268 (17.17) |  |  |
| **Difficulty initiating sleep** |  |  |  | 0.91 | 0.340 |
| Yes | 666 | 559 (83.93) | 107 (16.07) |  |  |
| No | 1,797 | 1,536 (85.48) | 261 (14.52) |  |  |
| **Nocturnal awakenings** |  |  |  | 0.18 | 0.673 |
| Yes | 921 | 787 (85.45) | 134 (14.55) |  |  |
| No | 1,542 | 1,308 (84.82) | 234 (15.18) |  |  |
| **Sleep duration** |  |  |  | 32.57 | ＜0.001^*^ |
| ＜6h | 339 | 286 (84.37) | 53 (15.63) |  |  |
| 6-8h | 1,657 | 1,450 (87.51) | 207 (12.49) |  |  |
| ≥8h | 467 | 359 (76.87) | 108 (23.13) |  |  |
| **Denture use** |  |  |  | 2.29 | 0.130 |
| Yes | 1,447 | 1,244 (85.97) | 203 (14.03) |  |  |
| No | 1,016 | 851 (83.76) | 165 (16.24) |  |  |
| **History of falls in the past year** |  |  |  | 6.39 | 0.011^*^ |
| Yes | 274 | 219 (79.93) | 55 (20.07) |  |  |
| No | 2,189 | 1,876 (85.70) | 313 (14.30) |  |  |
| **Fall frequency within the past year** |  |  |  | 10.19 | 0.006^*^ |
| 0 | 2,191 | 1,878 (85.71) | 313 (14.29) |  |  |
| 1～2 | 236 | 1,192 (81.36) | 44 (18.64) |  |  |
| ＞3 | 36 | 25 (69.44) | 11 (30.56) |  |  |
| **Fall-related injuries** |  |  |  | 5.16 | 0.023^*^ |
| With | 178 | 141 (79.21) | 37 (20.79) |  |  |
| Without | 2,285 | 1,954 (85.51) | 331 (14.49) |  |  |

**P*＜0.05

**TABLE S2 Prevalence of dementia by medical history.**

| Variables | Total | Normal | Dementia | $\chi^{2}$ | *P*-value |
| --- | --- | --- | --- | --- | --- |
| **Hypertension** |  |  |  | 6.90 | 0.009^*^ |
| Yes | 1,150 | 955 (83.04) | 195 (16.96) |  |  |
| No | 1,313 | 1,140 (86.82) | 173 (13.18) |  |  |
| **Diabetes** |  |  |  | 4.72 | 0.030^*^ |
| Yes | 499 | 409 (81.96) | 90 (18.04) |  |  |
| No | 1,964 | 1,689 (85.85) | 278 (14.15) |  |  |
| **Dyslipidemia** |  |  |  | 3.85 | 0.050^*^ |
| Yes | 189 | 170 (89.95) | 19 (10.05) |  |  |
| No | 2,274 | 1,925 (84.65) | 349 (15.35) |  |  |
| **Gout** |  |  |  | 0.00 | 1.000 |
| Yes | 87 | 74 (85.06) | 13 (14.94) |  |  |
| No | 2,376 | 2,021 (85.06) | 355 (14.94) |  |  |
| **Atherosclerosis^a^** |  |  |  | 0.96 | 0.493 |
| Yes | 16 | 15 (93.75) | 1 (6.25) |  |  |
| No | 2,447 | 2,080 (85.00) | 367 (15.00) |  |  |
| **Stroke** |  |  |  | 19.72 | ＜0.001^*^ |
| Yes | 98 | 68 (69.39) | 30 (30.61) |  |  |
| No | 2,365 | 2,027 (85.71) | 338 (14.29) |  |  |
| **Cardiovascular diseases** |  |  |  | 0.08 | 0.780 |
| Yes | 285 | 244 (85.61) | 41 (14.39) |  |  |
| No | 2,178 | 1,851 (84.99) | 327 (15.01) |  |  |
| **Chronic pulmonary diseases** |  |  |  | 0.31 | 0.578 |
| Yes | 42 | 37 (88.10) | 5 (11.90) |  |  |
| No | 2,421 | 2,058 (85.01) | 363 (14.99) |  |  |
| **Rheumatoid arthritis** |  |  |  | 0.01 | 0.969 |
| Yes | 34 | 29 (85.29) | 5 (14.71) |  |  |
| No | 2429 | 2,066 (85.06) | 363 (14.94) |  |  |
| **Connective tissue disorders** |  |  |  | 1.30 | 0.254 |
| Yes | 108 | 96 (88.89) | 12 (11.11) |  |  |
| No | 2,355 | 1,999 (84.88) | 356 (15.12) |  |  |
| **Chronic nephritis^a^** |  |  |  |  | 0.787 |
| Yes | 26 | 23 (88.46) | 3 (11.54) |  |  |
| No | 2,437 | 2,072 (85.02) | 365 (14.98) |  |  |
| **Liver diseases**** |  |  |  |  | 0.341 |
| Yes | 19 | 18 (94.74) | 1 (5.26) |  |  |
| No | 2,444 | 2,077 (84.98) | 367 (15.02) |  |  |
| **Chronic gastroenteritis** |  |  |  | 0.02 | 0.889 |
| Yes | 56 | 48 (85.71) | 8 (14.29) |  |  |
| No | 2,407 | 2,047 (85.04) | 360 (14.96) |  |  |
| **Splenic and gallbladder disorders^a^** |  |  |  |  | 0.376 |
| Yes | 10 | 10 (100.00) | 0 (0.00) |  |  |
| No | 2,453 | 2,085 (85.00) | 368 (15.00) |  |  |
| **Malignant tumors^a^** |  |  |  |  | 0.493 |
| Yes | 16 | 15 (93.75) | 1 (6.25) |  |  |
| No | 2,447 | 2,080 (85.00) | 367 (15.00) |  |  |
| **Visual impairment** |  |  |  | 0.27 | 0.606 |
| Yes | 89 | 74(83.15) | 15 (16.85) |  |  |
| No | 2,374 | 2,021(85.13) | 353 (14.87) |  |  |
| **Hearing impairment** |  |  |  | 8.05 | 0.005^*^ |
| Yes | 43 | 30 (69.77) | 13 (30.23) |  |  |
| No | 2,420 | 2,065 (85.33) | 355 (14.67) |  |  |
| **Depression** |  |  |  | 16.15 | ＜0.001^*^ |
| Yes | 48 | 31 (64.58) | 17 (35.42) |  |  |
| No | 2,415 | 2,064 (85.47) | 351 (14.53) |  |  |

**P*＜0.05；^a^The *P*-value was estimated by *Fisher's* exact test

**TABLE S3 Multicollinearity test.**

| **Variable** | **Multicollinearity between variables** | |
| --- | --- | --- |
|  | **Tolerance** | **VIF** |
| **Age** | 0.877 | 1.140 |
| **Education level** | 0.736 | 1.359 |
| **Occupation** | 0.756 | 1.323 |
| **Marital status** | 0.916 | 1.092 |
| **Physical exercise** | 0.948 | 1.054 |
| **Cognitive activity** | 0.929 | 1.077 |
| **Sleep duration** | 0.972 | 1.029 |
| **Fall frequency within the past year** | 0.461 | 2.167 |
| **Fall-related injuries** | 0.461 | 2.171 |
| **Hypertension** | 0.940 | 1.063 |
| **Diabetes** | 0.965 | 1.036 |
| **Dyslipidemia** | 0.990 | 1.010 |
| **Stroke** | 0.954 | 1.048 |
| **Hearing impairment** | 0.994 | 1.007 |
| **Depression** | 0.957 | 1.045 |

**TABLE S4 Hosmer–Lemeshow test.**

| **Step** | 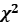 | ***df*** | ***P*** |
| --- | --- | --- | --- |
| 1 | 6.283 | 8 | 0.616 |
| 2 | 5.883 | 8 | 0.660 |
| 3 | 10.619 | 8 | 0.224 |
| 4 | 7.899 | 8 | 0.443 |
| 5 | 5.622 | 8 | 0.690 |
| 6 | 5.604 | 8 | 0.691 |
| 7 | 8.664 | 7 | 0.278 |
